# Virtual Reality Behavioral Activation as an Intervention for Major Depressive Disorder: Case Report

**DOI:** 10.2196/24331

**Published:** 2020-11-03

**Authors:** Margot Paul, Kim Bullock, Jeremy Bailenson

**Affiliations:** 1 PGSP-Stanford PsyD Consortium Palo Alto, CA United States; 2 Department of Psychiatry & Behavioral Sciences Stanford School of Medicine Stanford, CA United States; 3 Department of Communication Stanford University Stanford, CA United States

**Keywords:** virtual reality, case report, major depressive disorder, behavioral activation, VR, depression, COVID-19, behavior, intervention, feasibility, acceptability, telehealth, pilot study

## Abstract

**Background:**

Major depressive disorder (MDD) is a global problem with an increasing incidence and prevalence. There has additionally been an increase in depression due to the COVID-19 pandemic. Behavioral activation is considered an evidence-based treatment for MDD. However, there are many barriers that could hinder one’s ability to engage in behavioral activation, with COVID-19 “shelter-in-place” and social distancing orders being current and large impediments. Virtual reality (VR) has been successfully used to help treat a variety of mental health conditions, but it has not yet been used as a method of administering behavioral activation to a clinical population. Using VR to engage in behavioral activation could eliminate barriers that pandemic precautions place and help decrease symptoms of depression that are especially exacerbated in these times.

**Objective:**

The following case report examines the feasibility, acceptability, and tolerability of VR behavioral activation for an adult with MDD during a global pandemic. This participant was part of a larger pilot study, and the case serves as a description of the VR intervention.

**Methods:**

The participant engaged in a weekly 50-minute psychotherapy Zoom session for 4 weeks, in which a modified behavioral activation protocol was administered using a VR headset to simulate activities. Data on mood ratings, homework compliance, and headset use were obtained from the headset. Acceptability, tolerability, and depression symptoms were obtained using self-report rating scales.

**Results:**

The intervention was feasible, acceptable, and tolerable, as reported by this participant. The participant’s depressive symptoms decreased by five-points on the Patient Health Questionnaire-9 over a month, with a beginning score of 10 (moderate depression) and a final score of 5 (mild depression).

**Conclusions:**

The implications of these findings for future research are discussed.

**Trial Registration:**

ClinicalTrials.gov NCT04268316; http://clinicaltrials.gov/ct2/show/NCT04268316

## Introduction

Depression, classified as major depressive disorder (MDD) by the Diagnostic and Statistical Manual of Mental Disorders, 5th Edition (DSM-5), is a global problem with an increasing incidence and prevalence [1]. MDD is characterized by the experience of at least five of the following nine symptoms nearly every day during the same 2 week period (with at least one of the symptoms being either depressed mood or loss of interest or pleasure): depressed mood; diminished interest or pleasure in activities; significant weight loss when not dieting, weight gain, or a change in appetite; insomnia or hypersomnia; psychomotor agitation or retardation; fatigue; feelings of worthlessness or excessive and inappropriate guilt; difficulty concentrating; and suicidal thoughts [2]. As such, MDD is heterogeneous, given that two people can hold the diagnosis with only one overlapping symptom. However, regardless of an individual’s symptom presentation, those who meet diagnostic criteria experience significant distress or impairment in areas of functioning [2].

More than 322 million people worldwide experience symptoms of depression and about 788,000 people die each year from suicide, with suicide being the second global leading cause of death for people aged 15-29 years [1]. COVID-19 has further led to an increased risk for people developing depression worldwide, due to containment measures such as confinement to one’s home with “shelter-in-place” and community shutdown orders lasting for months [3,4]. As a result of these containment measures and their subsequent negative consequences on individuals, such as social isolation and increased rates of unemployment, there have been estimates that potentially 50,000 more individuals could commit suicide worldwide [5].

Depressive disorders are worldwide the “single largest contributor to non-fatal health loss” and are among the leading drivers for years lived with disability [1]. This latter metric accounts for the number of people who are affected by the disorder as well as the “severity and disability associated with their symptoms” [6]. Specifically, MDD is estimated to be the 11th leading cause of disability and mortality worldwide and the second leading cause of disability among all disease and injury in the United States [7,8]. Data analyzed from 36,309 US adults between 2012 and 2013 found that the lifetime prevalence of MDD was 20.6%, while a 1-year prevalence was 10.4% [9]. This means that, over a person’s lifetime, there is more than a one in five chance of having a major depressive episode. Among adults with MDD, about 64% had a severe impairment [10].

Given the severe impact that depressive symptoms have on individuals and society, it is imperative to identify effective treatment options. Many evidence-based treatments have been identified for MDD, behavioral activation (BA) being one of them [11]. BA is defined “as a structured, brief psychotherapeutic approach that aims to (a) increase engagement in adaptive activities (which often are those associated with the experience of pleasure or mastery), (b) decrease engagement in activities that maintain depression or increase risk for depression, and (c) solve problems that limit access to reward or that maintain or increase aversive control” [12]. This is in direct response to the behavioral theory of depression, which states that a dearth of response-contingent positive reinforcement catalyzes symptoms of depression due to less frequent engagement in pleasant activities or behavioral avoidance [12]. Thus, by helping people who have depression to become behaviorally activated through engaging in activities that they find pleasurable or that lead to a sense of accomplishment and mastery, they are able to regain the lost positive reinforcement and improve mood symptoms.

Despite BA’s effectiveness and ease of dissemination and use in primary care settings, obstacles exist to its implementation. First, finding the ongoing internal motivation to become behaviorally activated is not an easy task for people who struggle with depression, due to the nature of the symptoms themselves [12]. There may also be external obstacles that prevent engagement in pleasant activities, such as finances and mobility. For example, an individual may find pleasure in travel or an adrenaline-filled activity, which may be too costly to engage in or not available due to safety concerns from physical conditions. Another person may be unable to engage in activities they previously enjoyed, such as hiking or visiting distant places, due to mobility constraints, lack of social connections, and community or pandemic restrictions. As previously mentioned, the COVID-19 outbreak led to widespread confinement to one’s home with “shelter-in-place” and community shutdown orders lasting for months, preventing individuals from partaking in the activities they used to enjoy. Thus, it is vital to consider alternative treatment methods that patients may access and more easily engage in, especially for those that may be unable to receive in-person treatment.

The use of technology as an adjunct to or a method of delivering mental health treatments is becoming increasingly popular as a way to fill this treatment gap [13]. One technology medium, virtual reality (VR), is defined as a “computer-generated simulation, such as a set of images and sounds that represents a real place or situation, that can be interacted with, in a seemingly real or physical way by a person using special electronic equipment. It can transmit visual, auditory, and various sensations to users through a headset to make them feel as if they are in a virtual or imagined environment” [13]. VR has been successfully used to help treat a variety of mental health conditions, and the use of VR could help eliminate many of the aforementioned barriers to care due to a sense of presence that can match real-world activities [13-15]. Unlike engaging in real-world activities, VR is readily accessible and can consistently be used, making it a potentially beneficial therapeutic modality when other activities are barred.

Although there is minimal risk when using a VR headset, studies have indicated that the side effects may include cybersickness, often comprising three subscales: nausea (N), oculomotor (O), and disorientation (D). N includes increased salivation, sweating, nausea, upset stomach, or burping; O includes fatigue, headache, eyestrain, or difficulty focusing; D includes vertigo, dizziness, and blurred vision [16-18]. The cause of cybersickness is largely unknown, but there are many theories. One of the predominant hypotheses is that cybersickness is due to a mismatch between visual and vestibular cues [16,18]. In other words, the person using the VR headset is perceiving movement without feeling the movement or doing so themselves, causing feelings of sickness [16,18]. Research has illustrated that the rates of cybersickness increase with time wearing the headset [16]. Thus, although the exact cause of cybersickness remains unknown and can be unpleasant, there are precautions that can be taken to minimize the risk.

This case represents results from a single subject who was part of a larger pilot study currently being explored to test the feasibility, acceptability, and tolerability of using a VR headset as a way to administer BA during the COVID-19 pandemic. If pleasant activities can be successfully simulated and found effective using a VR headset, this would eliminate many obstacles to receiving care or engaging in pleasant activities such as cost-related impediments, ability-related obstacles, or other access-related difficulties such as the COVID-19 “shelter-in-place” orders. Given the plethora of VR options readily available online for free and the cheaper headset selections, VR is now more publicly accessible than in previous years [19]. If using VR to simulate pleasurable activities decreases the symptoms of depression, it could potentially provide relief for many people who would otherwise not be able to engage in such activities. This case hypothesized that VR BA would be an acceptable, feasible, and tolerable method of delivering a BA intervention for an individual diagnosed with MDD, and there would be a decrease in symptoms of depression after using the headset.

## Methods

### Materials and Apparatus

**Figure 1:**
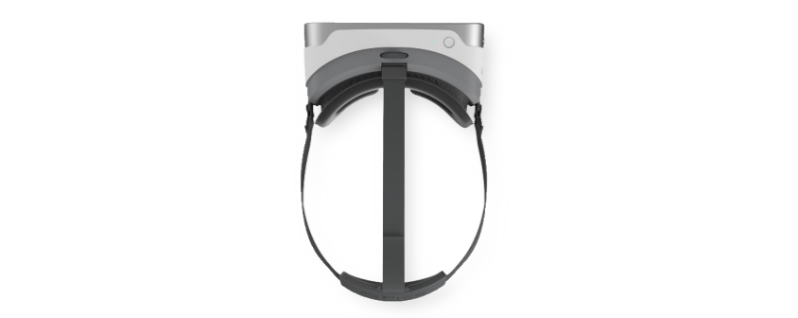
Virtual reality headset.

A VR headset supplied by Limbix, now partnered with BehaVR, was used. This headset had a 5.5-inch screen size with 2560 x 1440-pixel resolution, a screen aspect ratio of 16:9, a 92° field of view, 3 degrees of freedom, and a refresh rate of 70 Hz [20] (see Figure 1). This headset was chosen due to the fact that it was a wireless system with preprogrammed content that did not require the use of a phone or computer. The headset was easily turned on and off, and used the motion of the participant’s head to pinpoint the desired content with a visually simulated white circle that could then be clicked with a side button by one’s finger. The ease of use was imperative, due to the participant’s need to have the headset at home for engaging in activities between sessions. The Limbix devices were returned to the protocol director after study completion.

**Figure 2:**
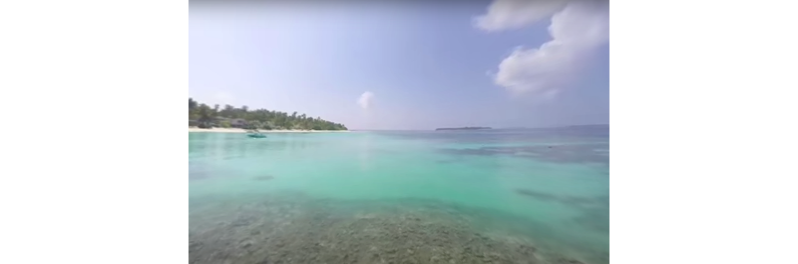
Screenshot of Visit Maldives.

**Figure 3:**
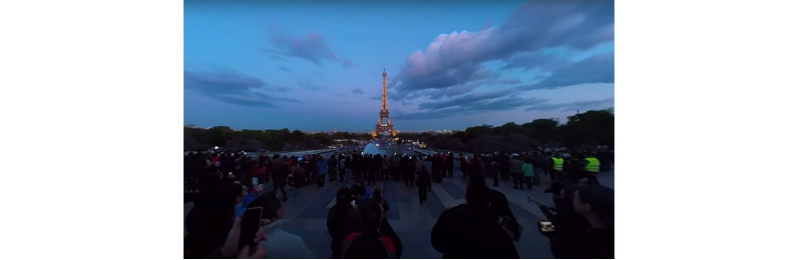
Screenshot of Paris.

**Figure 4:**
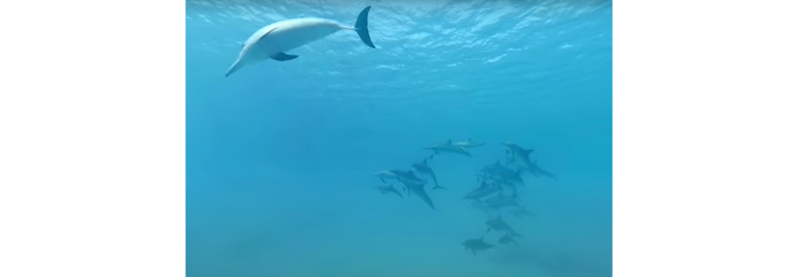
Screenshot of Swim with Dolphins.

**Figure 5:**
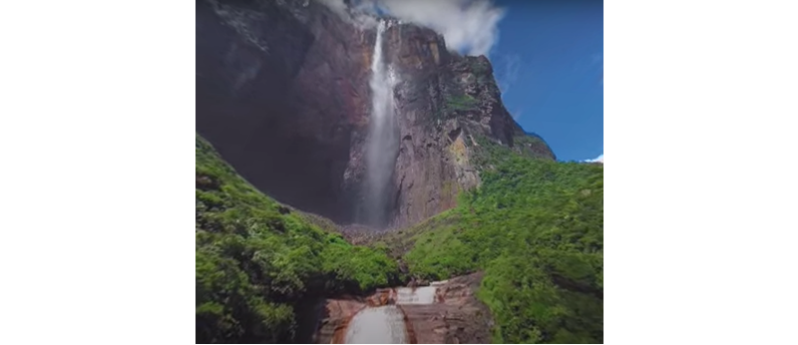
Screenshot of Angel Falls.

The immersive 360° videos were chosen from 360° videos already accessible on YouTube (see Multimedia Appendix 1). A total of 37 videos were selected based on activities from the Pleasant Events Schedule [21] and the subsequent quality of the available image (at least 4K resolution). These videos were uploaded onto the headset and each video was sorted into at least one of the five categories: animals; sports, dance, or arts; adrenaline; travel; and hiking or outdoors (see Multimedia Appendix 2). These categories were chosen to provide participants a diversity of options that most align with their values and interests. For example, a person experiencing symptoms of depression who previously enjoyed travel could explore the beauty of the Maldives (see Figure 2) or the majesty of the Eifel Tower in Paris (see Figure 3). Another participant may enjoy nature and could choose to experience swimming with dolphins (see Figure 4) or visiting a waterfall in Venezuela (see Figure 5). The videos ranged in length from 1 minute and 2 seconds to 10 minutes, to minimize the risk of cybersickness [16].

### Measures

#### Demographics

The participant was asked to confirm his name and date of birth during the intake assessment screening. During the intake visit, the participant completed a written demographic form over Zoom (Zoom Video Communications, Inc), which asked him to identify information about gender identity, racial identity, mental health treatment history, VR use history, and history of epilepsy and treatment (see Multimedia Appendix 3).

#### The Mini International Neuropsychiatric Interview

The Mini International Neuropsychiatric Interview (MINI) was verbally administered during the Zoom intake and was used to evaluate inclusion and exclusion criteria. It is a short diagnostic structured interview that assesses the 17 most common psychiatric disorders in the DSM-5. In a study comparing the validity and reliability of the MINI to the Structured Clinical Interview for DSM, it was found that the MINI had good reliability and validity, and took half the amount of time [22].

#### Mood

The participant’s mood was primarily measured using the Patient Health Questionnaire-9 (PHQ-9) at four timepoints and was verbally administered by the protocol director. The PHQ-9 is a nine-item self-report that measures an individual’s depression symptoms by mapping onto the nine criteria in the DSM-5 and rating them from 0 (not at all) to 3 (nearly every day), with a score range of 0-29, with 29 indicating the most severe depression and 0 indicating a lack of depression. A PHQ-9 score of 10 or higher indicates the presence of MDD with a sensitivity of 88% and a specificity of 88% [23]. The PHQ-9 has an excellent construct validity and a high internal reliability (Cronbach alpha .89) [23].

#### Presence

The intensity of presence felt in the virtual environment was adapted from the five-question telepresence scale outlined by Nowak and Biocca [24], which has an alpha of .88. The adapted scale used in this study incorporated three questions with five response choices ranging from “Not at all” to “Very Strongly.” Question 3 was modified from Nowak and Biocca’s [24] “To what extent did you feel immersed in the environment you saw/heard?” to “How much did it feel as if you visited another place?” to clarify the wording and make it more distinct from question 2. These questions were completed on a worksheet by the participant after each VR activity and securely emailed to the protocol director before each session.

#### Tolerability

Physical tolerability was measured using the Simulator Sickness Questionnaire (SSQ) [25]. This questionnaire includes 16 symptoms that each load differently onto the three clusters of sickness: oculomotor (O; eyestrain, difficulty focusing, blurred vision, headache; Cronbach alpha .91), disorientation (D; dizziness, vertigo; Cronbach alpha .88), and nausea (N; nausea, stomach awareness, increased salivation, burping; Cronbach alpha .84) [26]. Each symptom has a rating choice of “No more than usual” (0), “Slightly more than usual” (1), “Moderately more than usual” (2), or “Severely more than usual” (3). Emotional tolerability was measured by using the Brief Agitation Measure. This measure consists of three items with each item scored from “Strongly Disagree” (1) to “Strongly Agree” (7). This measure has a high internal consistency with a coefficient alpha of .91 [27]. These questions were completed on a worksheet by the participant after each VR activity and securely emailed to the protocol director before each session.

#### Acceptability

Acceptability was assessed using an adapted version of the technology acceptance model (TAM), a valid and reliable (Cronbach alpha ranging from .73 to .94) measure [28]. The TAM used in this study encompassed 13 questions with the subcategories of “Perceived Usefulness,” “Perceived Ease of Use,” “Attitudes Toward Use,” and “Intention to Use Technology.” Participants were given the option of circling one of five choices ranging from “Strongly Disagree” to “Strongly Agree.” These questions were completed on a worksheet by the participant after each VR activity and securely emailed to the protocol director before each session.

### Protocol

#### Recruitment

This case report is part of a current study that aims to continue recruitment until either 30 participants are enrolled or the timeline of January 15, 2021, whichever comes first. Participants were recruited from a study flyer posted at Stanford School of Medicine, Department of Psychiatry & Behavioral Sciences located at 401 Quarry, Palo Alto, CA. The flyer and description of the study were also listed on the Department of Psychiatry & Behavioral Sciences at Stanford University School of Medicine’s currently recruiting studies website and ClinicalTrials.gov. Individuals calling into Stanford Psychiatry’s intake team were also informed about the study and given the protocol director’s contact information, if interested. Curify, a health-technology startup, also assisted in recruitment by advertising the study on Facebook.

#### Screening (Part 1)

The participant who contacted the protocol director expressing interest in the study was scheduled for an initial phone screen, where he was briefly assessed for initial eligibility and provided with the opportunity to ask questions about the study. Initial eligibility was determined by a PHQ-8 score of 10 or greater [23] as well as a brief questionnaire that was designed to be the first preliminary screener for inclusion criteria (see Multimedia Appendix 4). After the participant met initial eligibility and stated that he was still interested in participation, a formal intake was scheduled via Zoom, due to COVID-19 restrictions, and he was securely emailed the consent form to review prior to meeting.

#### Screening (Part 2)

After reviewing any questions and securely emailing the signed consent form back to the protocol director, the intake session occurred. During the intake session, the participant was asked to verbally complete the demographic questionnaire while the protocol director shared her screen via Zoom. The participant was subsequently administered the MINI by the protocol director. The participant was then informed of his eligibility and was scheduled for his first session via Zoom.

#### Randomization

Before the first session, this participant was randomized into one of the three study arms using five opaque, concealed envelopes in sets of six to preserve balance throughout the study. The participant had a one in three chance of being randomized into each group.

#### Intervention: VR BA Arm

The VR BA study arm followed the protocol for brief BA based on the guidance of Funderburk et al [29] and Jacobson et al [30]. That is, the treatment incorporated the four component parts that Jacobson et al [30] outlined: establishing the therapeutic relationship, developing goals for treatment, conducting a functional analysis, and treatment review with relapse prevention.

Funderburk et al’s [29] brief treatment protocol assisted in outlining the flow of information per session, with the first session focusing on establishing rapport, identifying activities that the participant valued or felt a sense of mastery or pleasure in from the past, introducing the activity log, and setting activity goals. The second session focused on reviewing homework and the connection between mood and activities, addressing barriers and problem-solving, and scheduling new activity goals. The third and fourth sessions similarly reviewed materials, addressed barriers to completing goals and problem-solving these barriers, and created new activity goals.

Specifically, the participant in the VR BA arm of the study met with the protocol director once per week for 4 weeks over Zoom for 50 minutes to receive BA therapy. He was securely emailed the mood activity log, the VR list of activities, and the post-VR questionnaire prior to the first session. The VR headset was shipped to his address prior to the first session as well.

During the first session, psychoeducation around the connection between thoughts, behaviors, and feelings was discussed, and the cognitive behavioral therapy triangle was shown via screen sharing. The participant was then introduced to the idea of BA and briefly explained the theory behind the evidence outlined by Lewinsohn [31]. The protocol director explained the difference between pleasure and mastery activities, and mapped out the participant’s previous day, hour-by-hour, with him to determine how often he engaged in pleasurable or mastery activities. The participant was then asked to use the mood activity log and schedule, in session, at least four VR “activities” that he may enjoy into his upcoming week, as well as complete the log (see Multimedia Appendix 5).

The participant was then shown how to use the headset and asked to complete a short activity in VR during the session to ensure proper use of the headset. He was informed that he could move his head and body, but he should remain seated for his safety. The participant was asked to complete the post-VR questionnaire assessing spatial presence, simulator sickness, agitation, and acceptability every time he finished an activity in VR (see Multimedia Appendix 6). Additionally, the headset prompted him to rate his mood on a scale of 1-10 (1=worst ever felt; 10=best ever felt) before and after each activity. Barriers were anticipated and problem-solving strategies were discussed.

**Figure 6:**
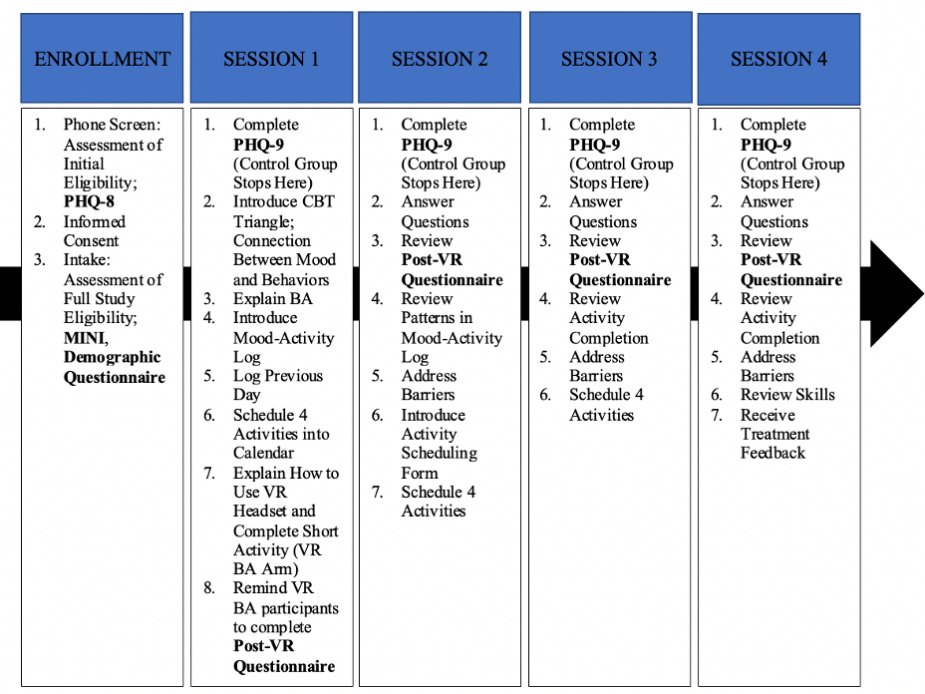
Study timeline. BA: behavioral activation; CBT: cognitive behavior therapy; MINI: Mini International Neuropsychiatric Interview; PHQ: Patient Health Questionnaire; VR: virtual reality.

During session two, the protocol director reviewed the mood activity log with the participant and checked-in regarding goal attainment. Barriers to completion of activities and problem-solving strategies were discussed. New activity goals were then introduced and scheduled in session using an activity scheduling form, which was securely emailed prior to session two (see Multimedia Appendix 7). Session three followed the same structure as session two, with review of the activity scheduling form instead of the mood activity log. During session four, the treatment and skills were reviewed, and feedback was attained (see Figure 6 for study timeline).

## Results

### Demographic and Background Information

This case report used the information and data gathered from the first participant who completed the VR BA arm of the study. This participant contacted the protocol director, stating that he was interested in participating in the study. After the initial phone screen and intake session were complete, he was informed that he met criteria to participate in the study. He was then randomized into the VR BA arm of the study.

The participant was a Caucasian male in his early 40s with a history of depression, who had been treated with psychotherapy and medication management. The participant stated that he had never used a VR device.

### Medications

The participant was taking Fluoxetine and Mirtazapine (dosages unknown) to treat his symptoms of depression.

### Psychiatric History

The participant stated that he had engaged in weekly talk therapy for years and was currently engaged in weekly psychotherapy for an hour and was being followed for medication management. The participant experienced six prior episodes of depression and met criteria for current MDD on the MINI. The participant denied current suicidal ideation or intent but stated that he had one previous suicide attempt over 10 years ago. The participant had a family history of bipolar disorder, with two paternal first cousins being diagnosed, but he denied ever experiencing any manic or hypomanic symptoms and did not meet criteria for bipolar disorder. The participant met criteria for mild cannabis use disorder, in early remission, given that he reported abstaining from cannabis for over 6 months. The participant additionally met criteria for bulimia nervosa, with about one inappropriate compensatory behavior a week after an eating binge.

### Medical History

The participant denied any history of seizures. He also denied any underlying medical illness.

### Mental Status Examination

During all meetings with the protocol director, the participant was alert and oriented to person, place, and time, and did not endorse any auditory or visual hallucinations. The participant did not appear to be in acute distress and denied suicidal ideation. He was appropriately dressed and well groomed. His speech, volume, and prosody were within normal limits. The participant’s affect was content congruent, and he was agreeable to the protocol director’s instruction and questioning.

### Intake and Session 1

During the initial phone session, the participant’s PHQ-8 score was a 10. The first session took place 9 days later and his PHQ-9 score was an 8. The subsequent three sessions took place exactly a week apart from each other.

### Session 2

At the beginning of session two, the participant’s PHQ-9 score decreased to a 7. During this session, the participant reflected that he enjoyed using the VR headset during the week because “VR can give [him] new experiences that [he] would not normally be able to do.” He also shared that using the VR headset had helped him to feel better, with the hypothesis that the “novelty helps with depression.” The participant further noted that he found himself “more motivated to do other things after using the headset.” He provided the feedback that the videos were not too short, and he liked that the length of each activity was provided; however, he thought that the motorcycle video was too long and repetitive at 10 minutes.

### Session 3

At the beginning of session three, the participant’s PHQ-9 score decreased to 6. During this session, the participant remarked that it was easier for him to engage in VR activities than activities in real life because he keeps the headset nearby, knows it is a short time commitment, and that he will feel better after using the headset. He further stated that after completing a VR activity, he experienced increased motivation to partake in a real life activity, enumerating his thought of “I may as well try something else” after using the headset. The participant also remarked that he used the VR headset to help replace activities he wanted to decrease, such as social media.

### Session 4

At the beginning of session four, the participant’s PHQ-9 score was 5. The participant stated that due to activity monitoring he was completing more activities than usual, both in VR and in real life. He also mentioned that using the VR headset provided him with a sense of accomplishment and something to look forward to. He attributed the overall 5-point decrease in his PHQ-9 scores to the fact that he had been increasing his activities, had more motivation after using the VR headset, and was feeling a greater sense of mastery. He stated that the appeal of using VR was the ability to gain exposure to new things, which provided the impetus for him to engage in novel activities in real life, such as visiting new parks. The participant remarked that although there were only 37 VR activities to choose from, he still felt that the experiences were novel since he could look in different directions during each activity. He recalled that the key to frequently using the device was keeping it close by so that he would remember to use it instead of other, less helpful activities. He noted that 5 days prior his psychiatrist increased his dosage of Fluoxetine but stated that he did not currently feel a difference and was informed that the effects would not be felt for “a week or two.”

### Feasibility, Acceptability, Tolerability

The feasibility, or degree to which VR could successfully be integrated into BA treatment, was measured by commenting on qualitative barriers to use observed. Barriers were assessed by rates of dropout, adverse events, the number of times the headset was used, and the level of presence felt in the headset. The level of presence was calculated on a scale of 0 (not at all) through 4 (very strongly) for each question; and with three questions, there was a possibility of yielding a score between 0 and 12. The average total presence for the participant was then calculated as 9.53 out of 12. The participant completed the study and did not report any adverse events during the study. Although full homework completion required using the VR headset a minimum of four times per week, yielding a minimum total of 12 times, this participant used the headset a total of 21 times, while completing 15 post-VR worksheets. The participant used the headset six times during his first week, 10 times during his second week, and five times during his third week. Although the participant engaged in 15 of the 37 potential activities, he chose to participate in “Cats in Living Room,” “Bungee Swinging Canyon,” “Rollercoaster,” “Motorcycle Race,” “Swimming with Sharks,” and “Elephant” twice each, while engaging in the other nine activities once.

Acceptability of the VR BA treatment was measured using the TAM, with the agreeance choice on the Likert scale represented from 0 (Strongly Disagree) through 4 (Strongly Agree). The number of questions in each category determined the outcome range, which were then averaged. The participant maintained the same agreeance choices on all 15 questionnaires, indicating “Strongly Agree” for “Perceived Ease of Use” (average score: 12 out of 12), “Attitudes Towards Use” (average score: 16 out of 16), and “Intention to Use Technology” (average score: 12 out of 12), while in the “Perceived Usefulness” category, he rated “Strongly Agree” for questions 2 and 3 but “Agree” for question 1 (average score: 11 out of 12).

Physical tolerability of the VR headset was assessed by using the SSQ, and the emotional tolerability of the VR headset was assessed by using the Brief Agitation Measure. Physical tolerability was broken into each item and scaled from 0 (No more than usual) to 3 (Severely more than usual) for each item. The participant endorsed symptoms of *nausea*, *general discomfort*, *stomach awareness*, *sweating*, *increased salivation*, *vertigo*, *burping*, and *dizzy* (*eyes open*; see Table 1). Specifically, the participant endorsed the resulting symptoms after participating in the following four activities: “Cats in Living Room” (nausea), “Motorcycle Race” (nausea, general discomfort, stomach awareness, sweating, increased salivation, vertigo, dizzy [eyes open]), “Rollercoaster” (nausea, general discomfort, stomach awareness, sweating, increased salivation, vertigo, dizzy [eyes open]), and “Skiing” (nausea).

The total scores for physical tolerability were then summed, yielding a total score of 1.8 out of a potential 48, indicating high physical tolerability. Emotional tolerability was scored from 1 (Strongly Disagree) to 7 (Strongly Agree) per question; and with three questions, there was a possibility of yielding a score between 3 and 21. The average emotional tolerability score for this participant was a 3, indicating high emotional tolerability.

**Table 1:** Physical tolerability.

Measure (range of score)	Score, mean
Nausea (0-3)	0.33
General discomfort (0-3)	0.2
Stomach awareness (0-3)	0.27
Sweating (0-3)	0.27
Increased salivation (0-3)	0.13
Vertigo (0-3)	0.4
Burping (0-3)	0
Difficulty concentrating (0-3)	0
Difficulty focusing (0-3)	0
Eyestrain (0-3)	0
Fatigue (0-3)	0
Headache (0-3)	0
Blurred vision (0-3)	0
Dizzy (eyes open; 0-3)	0.2
Dizzy (eyes closed; 0-3)	0
Fullness of head (0-3)	0

## Discussion

### Principal Findings

This case demonstrates that VR BA was a feasible, acceptable, and tolerable method of delivering BA for an individual diagnosed with MDD during the COVID-19 pandemic. It also describes in detail the intervention that is being studied in a pilot randomized controlled trial (RCT) to be completed in 2021.

The participant used the headset more than was required for homework, did not verbally report any adverse events, and experienced an average presence of nearly 80% while using the headset. Although this is a relatively high presence rating, the authors conjecture that it was not higher for two reasons. First, the Limbix headset created a subtle effect that one is looking at the image through a screen, due to the simple device technology. Second, in using a 360° video, to give the illusion of movement, the image moves while the participant remained still, rather than the participant being able to walk around the virtual environment. It is possible that with a more advanced device, the presence rating would be higher. Still, his presence ratings were not correlated with his pre-VR to post-VR mood ratings.

The largest feasibility issue was working through COVID-19 “shelter-in-place” and transitioning the study from an in-person to telehealth design. However, the issue was solved by shipping the VR headset to the participant and using Zoom for the session meetings. Thus, the participant was still able to engage in weekly face-to-face sessions with the protocol director.

The participant rated the use of the headset as highly acceptable, giving the highest ratings of acceptability to all but one question. He indicated that using the headset was emotionally tolerable, denying any symptoms of agitation. He also rated the use of the headset as largely physically tolerable, providing a rating of about 3.8% intolerability. Although the participant did endorse varying degrees of nausea (N), general discomfort, stomach awareness (N), sweating (N), increased salivation (N), vertigo (D), and dizziness (eyes open; D) during three of the adrenaline activities and one nonadrenaline activity, the participant did not discuss these symptoms during sessions and still rated his post-VR mood as the same as or higher than his pre-VR mood after each of the adrenaline activities [16]. Given that the majority of his cybersickness symptoms occurred during the adrenaline activities of riding a rollercoaster, skiing, and riding a motorcycle, the authors hypothesize that this was a result of the mismatch between his vestibular and visual cues, since the movement of the image during adrenaline activities happens more quickly than when watching a sunset or observing nature [16,18]. Interestingly, despite endorsing symptoms of cybersickness during “Cats in Living Room,” “Rollercoaster,” and “Motorcycle Race” in week 1, he chose to engage in those three activities again during week 2. The activity of “Skiing” was participated in once, during his last week (week 3).

The participant experienced a 5-point decrease in depression symptoms on the PHQ-9 over a month, providing an initial rating of 10, which indicated moderate depression, and a final rating of 5, indicating very mild depression [23]. This decrease is clinically significant and illustrated that, despite the restrictions in place due to COVID-19, an individual was able to decrease his symptoms of depression using VR BA. He attributed this decrease in depression symptoms to increasing the number of his real life and virtual activities, a hypothesis that is in accordance with the behavioral theory of depression [12]. However, without a powered RCT, this finding could be due to a placebo effect. This is because the participant’s PHQ-9 score decreased from 10 during the initial intake to 8 at the beginning of the first session, before any intervention was provided. It should also be noted that the participant increased the dosage of his antidepressant medication simultaneously, adding a confound. However, the medication adjustment was done in between sessions 3 and 4, and there was already a downward trend of his PHQ-9 scores.

The strength of this VR BA intervention is that the mood ratings, activities completed, and amount of times using the headset were captured objectively and were standardized on the Limbix headset. Consequently, accurate home practice measurements were made, and the possibility of inaccurate homework reporting was eliminated. Additionally, this study took place during COVID-19 “shelter-in-place,” when real life activities were limited. The fact that this participant experienced mood increases after using the headset provides some evidence and possible potential for using VR to increase mood when real life activities are limited or treatment is being delivered remotely through telehealth.

This case study has several limitations. First, many of the quantitative and qualitative measures were subjective and completed by the participant. Although the participant engaged with the headset 21 times, he only completed 15 post-VR questionnaires, and thus, the complete data set was not able to be analyzed after every activity. Qualitative data collection that included further context was needed and is an important consideration for future research. In addition, given that this is a case report on one individual, the results may not be generalizable or help us identify causality. Results may not be applicable to all populations struggling with symptoms of depression, due to the heterogeneity of the disorder. Specifically, the participant began treatment with moderate symptoms of depression, and although BA has been shown to be effective for those with more severe symptoms, it is unknown whether using a VR headset to perform BA would yield these findings [12]. Unlike BA in real life, the participant was limited to the 37 activities on the headset, which were chosen primarily based on quality of image. Additionally, there was no follow-up, and thus, it is unknown whether the mood gains were lasting. Last, the participant had to return the Limbix headset to the protocol director upon study completion, and although there are low-cost VR options that the participant was educated to use, it is unknown if they will yield the same outcomes or compliance.

### Conclusion

To the authors’ knowledge, this was the first reported use of VR to administer BA for a person with MDD. This is a retrospective case report that used the data from the first VR BA participant in a larger, three-arm study. This case was a combination of VR1 and VR2 methodologies, as outlined by Birckhead et al [32], since it both discussed in-depth user feedback of the prototype VR intervention, and it also evaluated the feasibility, acceptability, and tolerability of the VR intervention for a participant diagnosed with MDD in a clinical setting. We believe that these findings will inspire other researchers to investigate and explore the use of VR BA as a method of treating individuals diagnosed with MDD. We also believe that these encouraging findings may inspire other researchers to pursue VR3 trials (powered RCT) to compare outcomes between using VR to administer BA and a control condition for individuals diagnosed with MDD [32].

## Appendix

Multimedia Appendix 1 Video URLs.

Multimedia Appendix 2 List of virtual reality activities.

Multimedia Appendix 3 Demographic questionnaire.

Multimedia Appendix 4 Telephone screen questions.

Multimedia Appendix 5 Activity monitoring form.

Multimedia Appendix 6 Post–virtual reality questionnaire.

Multimedia Appendix 7 Activity scheduling form.

## Abbreviations

BA: behavioral activation
D: disorientation
DSM-5: Diagnostic and Statistical Manual of Mental Disorders, 5th Edition
MDD: major depressive disorder
MINI: Mini International Neuropsychiatric Interview
N: nausea
O: oculomotor
PHQ-9: Patient Health Questionnaire-9
RCT: randomized controlled trial
SSQ: Simulator Sickness Questionnaire
TAM: technology acceptance model
VR: virtual reality
