# Supplementary material for: Virtual Reality Behavioral Activation as an Intervention for Major Depressive Disorder: Case Report
Source: JMIR Ment Health. 2020 Nov 3;7(11):e24331. doi: 10.2196/24331 (PMC7641650; doi:10.2196/24331)
Supplement: Multimedia Appendix 1 [file mental_v7i11e24331_app1.docx]

| **VR Videos** | **URL** | **Start Time (if different than URL)** | **End Time (if different than URL)** |
| --- | --- | --- | --- |
| Angel Falls | <https://www.youtube.com/watch?v=L_tqK4eqelA> | 00:10 | 06:10 |
| Antarctica | <https://www.youtube.com/watch?v=KVeSDRsrpUo> | 00:48 | 06:00 |
| Beach Cliff and Rocks | <https://vimeo.com/behavr/review/465971516/d44f1fa9d7?sort=lastUserActionEventDate&direction=desc> |  |  |
| Beach Sunrise | <https://vimeo.com/behavr/review/465470406/4c79cae64d?sort=lastUserActionEventDate&direction=desc> |  |  |
| Beach Walk | <https://vimeo.com/behavr/review/465969085/87e3eea024?sort=lastUserActionEventDate&direction=desc> |  |  |
| Bungee Swinging Canyon | <https://www.youtube.com/watch?v=tl6rVm9vx2g> | 00:10 | 01:39 |
| Cats in Living Room | <https://vimeo.com/behavr/review/465976504/7a2672fdd2?sort=lastUserActionEventDate&direction=desc> |  |  |
| Celestial Sphere | <https://www.youtube.com/watch?v=sDkQVPfrG8A> | 00:30 | 05:00 |
| Colorado Lake | <https://vimeo.com/behavr/review/465471739/9d8a1b04d0?sort=lastUserActionEventDate&direction=desc> |  |  |
| Elephant | <https://www.youtube.com/watch?v=mlOiXMvMaZo&t=25s> |  |  |
| Explore A Coral Reef | <https://www.youtube.com/watch?v=v64KOxKVLVg> | 00:05 | 05:15 |
| Explore the World | <https://www.youtube.com/watch?v=1_ifgJqLqTY> |  | 02:55 |
| Going to The Beach Sunset | <https://vimeo.com/behavr/review/465975013/7a1b31d98c?sort=lastUserActionEventDate&direction=desc> |  |  |
| Grand Canyon | <https://www.youtube.com/watch?v=WKAgKlnfkDA> |  | 02:25 |
| Half Moon Bay Sunset | <https://vimeo.com/behavr/review/465972737/ae993817ef?sort=lastUserActionEventDate&direction=desc> | 00:10 | 02:05 |
| Hamilton | <https://www.youtube.com/watch?v=k9AyO8h2I0k> |  |  |
| Hiking the Grand Canyon | <https://vimeo.com/behavr/review/465919232/f24755223c?sort=lastUserActionEventDate&direction=desc> | 22:18 | 33:00.0 |
| Horseback Riding Bermuda | <https://www.youtube.com/watch?v=zJolGInDb1M&t=101s> | 00:14 | 03:15 |
| London | <https://www.youtube.com/watch?v=SNx8B_oE8IY> | 00:10 | 03:04 |
| Machu Picchu | <https://www.youtube.com/watch?v=kl6ZXH1X4sc> | 00:24 |  |
| Motorcycle Race | <https://vimeo.com/behavr/review/465877949/fdd82a9ce9?sort=lastUserActionEventDate&direction=desc> |  | 10:00 |
| Northern Lights | <https://www.youtube.com/watch?v=A0C5kiFtmjI> | 00:10 | 04:45 |
| Paris | <https://www.youtube.com/watch?v=EkshFcLESPU> | 00:10 | 04:25 |
| Puppies | <https://www.youtube.com/watch?v=5qmmms4VP2k> |  |  |
| Rollercoaster | <https://www.youtube.com/watch?v=-xNN-bJQ4vI> | 00:10 | 01:52 |
| Skateboard Ramp | <https://www.youtube.com/watch?v=E07KdXQ34hk> | 00:10 | 01:20 |
| Skiing | <https://vimeo.com/behavr/review/465980732/74901933b9?sort=lastUserActionEventDate&direction=desc> |  |  |
| Starry Night | <https://www.youtube.com/watch?v=wCbR88NbziY> |  |  |
| Swim with Dolphins | <https://www.youtube.com/watch?v=BbT_e8lWWdo> | 00:26 | 01:30 |
| Swimming with Sharks | <https://www.youtube.com/watch?v=rG4jSz_2HDY&t=8s> | 00:06 | 01:55 |
| Switzerland | <https://www.youtube.com/watch?v=lhiNnWglMTM&t=7s> | 00:14 | 03:00 |
| Visit Maldives | <https://www.youtube.com/watch?v=MgJITGvVfR0> | 00:05 | 02:10 |
| Water Scenes | <https://www.youtube.com/watch?v=7AkbUfZjS5k> | 00:10 | 05:30 |
| White Lions | <https://www.youtube.com/watch?v=14O7AxqjiVY&t=73s> | 02:00 | 05:00 |
| Wild Animals Africa Sunset | <https://vimeo.com/behavr/review/465992792/15f6630ad6?sort=lastUserActionEventDate&direction=desc> | 01:00 | 12:00 |
| Wild Animals Baboons | <https://vimeo.com/behavr/review/465981652/02cd217d75?sort=lastUserActionEventDate&direction=desc> |  | 02:50 |
| Wild Animals Monkeys | <https://vimeo.com/behavr/review/465985612/73809b1ac5?sort=lastUserActionEventDate&direction=desc> |  | 01:41 |
