# Supplementary material for: Virtual Reality Behavioral Activation as an Intervention for Major Depressive Disorder: Case Report
Source: JMIR Ment Health. 2020 Nov 3;7(11):e24331. doi: 10.2196/24331 (PMC7641650; doi:10.2196/24331)
Supplement: Multimedia Appendix 2 [file mental_v7i11e24331_app2.docx]

**LIST OF VR ACTIVITIES**

VR activities - 37 total

Animals

- Cats in Living Room – 1:06
  - Watch as kittens play in a living room
- Wild Animals Baboons – 2:50
  - Observe baboons as they eat and play in the wild
- Swimming with Sharks – 1:55
  - Join scuba divers as they swim with sharks
- Wild Animals Monkeys – 1:41
  - Watch as wild monkeys play with and eat a watermelon
- Wild Animals Africa Sunset – 11:00
  - Observe the wild animals and scenery of the African plains from dawn through sunset.
- Elephant – 2:24
  - Watch as a herd of elephants walk by
- Puppies – 1:56
  - Watch as puppies play, eat, and sleep and listen as a narrator explains the life of puppies training to be a seeing-eye-dog.
- Explore A Coral Reef – 5:10
  - Explore a coral reef with a diver before coming to land and watching turtles hatch.
- Swim with Dolphins – 1:04
  - Swim with a pod of dolphins
- White Lions – 3:00
  - Observe white lions explore their surroundings.

Sports/Dance/Arts

- Horseback Riding Bermuda – 3:02
  - Join a family as they explore Bermuda on horseback
- Skiing – 1:53
  - Join a slalom skier as she zooms down the mountain
- Swimming with Sharks – 1:55
  - Join scuba divers as they swim with sharks
- Hamilton – 1:34
  - Watch the Hamilton cast rehearse *Wait for It*
- Starry Night – 2:32
  - Enter into Vincent van Gogh’s *Starry Night*
- Explore A Coral Reef – 5:10
  - Explore a coral reef with a diver before coming to land and watching turtles hatch.
- Swim with Dolphins – 1:04
  - Swim with a pod of dolphins

Adrenaline

- Bungee Swinging Canyon – 1:31
  - Bungee jump off of the Grand Canyon
- Rollercoaster – 1:43
  - Go for a ride in a rollercoaster
- Motorcycle Race – 10:00
  - Join a motorcyclist speed around a track
- Skateboard Ramp – 1:12
  - Join as a skateboarder attempts to skateboard down an extreme ramp
- Skiing – 1:53
  - Join a slalom skier as she zooms down the mountain

Travel

- Horseback Riding Bermuda –3:02
  - Join a family as they explore Bermuda on horseback
- Grand Canyon – 2:25
  - Explore the depths of the Grand Canyon largely by boat
- Switzerland – 2:46
  - Explore the scenery of Switzerland at night from the mountains.
- Machu Picchu – 2:17
  - Take a guided tour of Machu Picchu
- Antarctica – 5:12
  - Visit the glaciers of Antarctica and the penguins who live there
- Explore the World – 2:55
  - Take a brief trip around the world to explore some highlights.
- London – 2:54
  - Visit some of London’s most famous sights
- Paris – 4:15
  - Visit some of Paris’ most famous sights.
- Visit Maldives – 2:05
  - Visit a beautiful resort in the Maldives.

Hiking/Outdoors

- Water Scenes – 5:30
  - Experience various water scenes around the world, ranging from beaches to waterfalls.
- Beach Walk – 2:19
  - Take a walk around a beautiful beach.
- Beach Cliff and Rocks – 1:33
  - Travel to a rocky beach during the day and hear the ocean breeze.
- Half Moon Bay Sunset – 1:57
  - Watch a beach sunset amidst the cliffs of Half Moon Bay.
- Going to The Beach Sunset – 2:00
  - Watch a sunset while standing on a beach.
- Hiking the Grand Canyon – 10:42
  - Join a hiker as he travels through the Grand Canyon
- Beach Sunrise – 1:02
  - Watch a sunrise standing on a beach.
- Colorado Lake – 1:06
  - Enjoy the serene beauty of a lake.
- Angel Falls – 6:00
  - Experience a narrated tour of Angel Falls in Venezuela.
- Celestial Sphere – 7:50
  - Explore a starry night sky.
- Northern Lights – 4:35
  - Observe the aurora borealis amidst the mountains.
