# Supplementary material for: Virtual Reality Behavioral Activation as an Intervention for Major Depressive Disorder: Case Report
Source: JMIR Ment Health. 2020 Nov 3;7(11):e24331. doi: 10.2196/24331 (PMC7641650; doi:10.2196/24331)
Supplement: Multimedia Appendix 3 [file mental_v7i11e24331_app3.docx]

**DEMOGRAPHIC QUESTIONNAIRE**

1. Name:  __________________
2. What is your date of birth? _____________
3. What gender do you identify as?
   1. Female
   2. Male
   3. Transgender
   4. Non-binary/third gender
   5. Prefer not to say
   6. Other
4. What is your racial background?
   1. African American
   2. Black
   3. Chinese
   4. Other Pacific Islander
   5. Indian
   6. Japanese
   7. Korean
   8. Southeast Asian
   9. White Caucasian – Non-Hispanic
   10. Hispanic or Latino
   11. Mexican
   12. American Indian
   13. Alaskan Native
   14. Hawaiian Native
   15. Middle Eastern
   16. More than one race
   17. Unknown or not reported
   18. Decline to answer
5. Have you received mental health treatment(s) in the past?
   1. Yes: ________________
   2. No
6. Which, if any, of the following mental health treatment(s) are you receiving? How often?
   1. Counseling: ____________________
   2. Psychotropic medication: ________________
   3. None
   4. Other: please write _______________________
7. How many times have you used VR before?
   1. This is my first-time using VR
   2. 1 – 4
   3. 5 – 9
   4. 10+
8. In what capacity have you used VR in the past?
   1. Gaming: _______________
   2. Treatment: _______________
   3. Research: _________________
   4. Other: _________________
   5. N/A
9. Have you been diagnosed with epilepsy?
   1. Yes
      1. If yes, are you currently receiving treatment for it? Y N (please circle)
   2. No
10. Have you experienced any seizure(s) in the past 6 months?
    1. Yes
    2. No
