# Supplementary material for: Virtual Reality Behavioral Activation as an Intervention for Major Depressive Disorder: Case Report
Source: JMIR Ment Health. 2020 Nov 3;7(11):e24331. doi: 10.2196/24331 (PMC7641650; doi:10.2196/24331)
Supplement: Multimedia Appendix 4 [file mental_v7i11e24331_app4.docx]

Telephone Screen Questions

1. What is your name? ________________________
2. When is your birthday? _______________________
3. Do you feel comfortable speaking and understanding English? ___________________
4. Have you ever been diagnosed with psychosis or bipolar disorder? __________________
5. Have you experienced any seizures in the past 6 months? ________________________
6. If yes, are your seizures currently being treated? ______________________

Ask PHQ-8
