# Supplementary material for: Virtual Reality Behavioral Activation as an Intervention for Major Depressive Disorder: Case Report
Source: JMIR Ment Health. 2020 Nov 3;7(11):e24331. doi: 10.2196/24331 (PMC7641650; doi:10.2196/24331)
Supplement: Multimedia Appendix 5 [file mental_v7i11e24331_app5.docx]

**ACTIVITY MONITORING FORM**

|  | **Sunday** | **Monday** | **Tuesday** | **Wednesday** | **Thursday** | **Friday** | **Saturday** |
| --- | --- | --- | --- | --- | --- | --- | --- |
| 8:00 A.M. |  |  |  |  |  |  |  |
| 9:00 A.M. |  |  |  |  |  |  |  |
| 10:00 A.M. |  |  |  |  |  |  |  |
| 11:00 A.M. |  |  |  |  |  |  |  |
| 12:00 P.M. |  |  |  |  |  |  |  |
| 1:00 P.M. |  |  |  |  |  |  |  |
| 2:00 P.M. |  |  |  |  |  |  |  |
| 3:00 P.M. |  |  |  |  |  |  |  |
| 4:00 P.M. |  |  |  |  |  |  |  |
| 5:00 P.M. |  |  |  |  |  |  |  |
| 6:00 P.M. |  |  |  |  |  |  |  |
| 7:00 P.M. |  |  |  |  |  |  |  |
| 8:00 P.M. |  |  |  |  |  |  |  |
| 9:00 P.M. |  |  |  |  |  |  |  |
| **Overall Mood**  **(0 - 10)** |  |  |  |  |  |  |  |

Instructions: Please list the activities you did on each day of the week, whatever it may have been. Below each activity, rate the degree of pleasure (P) and mastery/accomplishment (M) on a 0-10 scale for each, where 0 is no pleasure or mastery/accomplishment and 10 is the greatest degree of pleasure or mastery/accomplishment Please also rate your **overall** mood for the entire day on a scale of 0 -10, where 0 is feeling the **worst** you could imagine feeling and 10 is feeling the **best** you could imagine feeling.
