# Supplementary material for: Virtual Reality Behavioral Activation as an Intervention for Major Depressive Disorder: Case Report
Source: JMIR Ment Health. 2020 Nov 3;7(11):e24331. doi: 10.2196/24331 (PMC7641650; doi:10.2196/24331)
Supplement: Multimedia Appendix 6 [file mental_v7i11e24331_app6.docx]

Post-VR Questionnaire

Date: _________________

What activity are you rating? ___________________

*Presence*

1. **To what extent did you feel like you were actually inside the virtual experience?**

Not at all            Slightly Moderately           Strongly Very Strongly

1. **To what extent did you feel surrounded by the virtual world you saw?**

Not at all            Slightly Moderately           Strongly Very Strongly

1. **How much did it feel as if you visited another place?**

Not at all            Slightly Moderately           Strongly Very Strongly

Please fill in the below questionnaire. Circle the description that best describes the severity of the specified symptom compared to **your baseline.** For example, if you are normally slightly fatigued, and this experience made you no more fatigued than usual, you would answer *no more than usual*. If this experience made you moderately more fatigued that normal, than you would answer *moderately more than usual.*

| **Nausea** | No more  than usual | Slightly more than usual | Moderately more than usual | Severely more than usual |
| --- | --- | --- | --- | --- |
| **General**  **discomfort** | No more  than usual | Slightly more than usual | Moderately more than usual | Severely more than usual |
| **Stomach awareness** | No more  than usual | Slightly more than usual | Moderately more than usual | Severely more than usual |
| **Sweating** | No more  than usual | Slightly more than usual | Moderately more than usual | Severely more than usual |
| **Increased**  **salivation** | No more  than usual | Slightly more than usual | Moderately more than usual | Severely more than usual |
| **Vertigo** | No more  than usual | Slightly more than usual | Moderately more than usual | Severely more than usual |
| **Burping** | No more  than usual | Slightly more than usual | Moderately more than usual | Severely more than usual |
| **Difficulty concentrating** | No more  than usual | Slightly more than usual | Moderately more than usual | Severely more than usual |
| **Difficulty**  **focusing** | No more  than usual | Slightly more than usual | Moderately more than usual | Severely more than usual |
| **Eyestrain** | No more  than usual | Slightly more than usual | Moderately more than usual | Severely more than usual |
| **Fatigue** | No more  than usual | Slightly more than usual | Moderately more than usual | Severely more than usual |
| **Headache** | No more  than usual | Slightly more than usual | Moderately more than usual | Severely more than usual |
| **Blurred vision** | No more  than usual | Slightly more than usual | Moderately more than usual | Severely more than usual |
| **Dizzy (eyes open)** | No more  than usual | Slightly more than usual | Moderately more than usual | Severely more than usual |
| **Dizzy (eyes closed)** | No more  than usual | Slightly more than usual | Moderately more than usual | Severely more than usual |
| **Fullness of head** | No more  than usual | Slightly more than usual | Moderately more than usual | Severely more than usual |

**Agitation Measure:**

Please read each item below and indicate to what extent you feel the statement describes you. Rate each statement on the scale below.

|  | Strongly  Disagree  (1) | Disagree  (2) | Somewhat  Disagree  (3) | Neither Agree nor Disagree  (4) | Somewhat  Agree  (5) | Agree  (6) | Strongly  Agree  (7) |
| --- | --- | --- | --- | --- | --- | --- | --- |
| I want to crawl out of my skin  after using the VR headset | 1 | 2 | 3 | 4 | 5 | 6 | 7 |
| I feel so stirred up inside I want to scream after using the VR headset | 1 | 2 | 3 | 4 | 5 | 6 | 7 |
| I feel a lot of emotional turmoil in my gut after using the VR headset. | 1 | 2 | 3 | 4 | 5 | 6 | 7 |

**Technology Acceptance Model (TAM):**

*Perceived Usefulness*

1. **Using the VR system would encourage me to do things I wouldn’t normally do**

Strongly Disagree Disagree Neutral Agree Strongly Agree

1. **Using the VR system would give me something to look forward to during the day**

Strongly Disagree Disagree Neutral Agree Strongly Agree

1. **I feel the VR system is useful**

Strongly Disagree Disagree Neutral Agree Strongly Agree

*Perceived Ease of Use*

1. **I feel the VR system is easy to use**

Strongly Disagree Disagree Neutral Agree Strongly Agree

1. **Learning to use the VR system would be easy for me**

Strongly Disagree Disagree Neutral Agree Strongly Agree

1. **My interaction with the VR system would be clear and understandable**

Strongly Disagree Disagree Neutral Agree Strongly Agree

*Attitudes Toward Use*

1. **I like the idea of using this VR system to engage in enjoyable activities**

Strongly Disagree Disagree Neutral Agree Strongly Agree

1. **I have a generally favorable attitude toward using this VR system**

Strongly Disagree Disagree Neutral Agree Strongly Agree

1. **I believe it is a good idea to use this system as part of my treatment process**

Strongly Disagree Disagree Neutral Agree Strongly Agree

1. **I am satisfied with the VR system**

Strongly Disagree Disagree Neutral Agree Strongly Agree

*Intention to Use Technology*

1. **If it were made available to me, I intend to use the VR system**

Strongly Disagree Disagree Neutral Agree Strongly Agree

1. **If it were made available to me, I would continue to use the VR system after completion of this study**

Strongly Disagree Disagree Neutral Agree Strongly Agree

1. **I would adopt the VR system in the future**

Strongly Disagree Disagree Neutral Agree Strongly Agree
