# Supplementary material for: Virtual Reality Behavioral Activation as an Intervention for Major Depressive Disorder: Case Report
Source: JMIR Ment Health. 2020 Nov 3;7(11):e24331. doi: 10.2196/24331 (PMC7641650; doi:10.2196/24331)
Supplement: Multimedia Appendix 7 [file mental_v7i11e24331_app7.docx]

Please rate your mood from 1-10 **before** and **after** each activity (1=worst ever felt; 10=best ever felt)

|  | **Week of:** | | | | | | |
| --- | --- | --- | --- | --- | --- | --- | --- |
| **Pleasant/Mastery Events** | Monday | Tuesday | Wednesday | Thursday | Friday | Saturday | Sunday |
| **1.** |  |  |  |  |  |  |  |
| **2.** |  |  |  |  |  |  |  |
| **3.** |  |  |  |  |  |  |  |
| **4.** |  |  |  |  |  |  |  |
| **5.** |  |  |  |  |  |  |  |
| **6.** |  |  |  |  |  |  |  |
| **7.** |  |  |  |  |  |  |  |
| **Total Activities Complete** |  |  |  |  |  |  |  |
| **Overall Mood (1-10)** |  |  |  |  |  |  |  |
